# Evaluation of candidate template beam models for a matched TrueBeam treatment delivery system

**DOI:** 10.1002/acm2.13278

**Published:** 2021-05-25

**Authors:** Jon B. Hansen, Sean P. Frigo

**Affiliations:** ^1^ Department of Human Oncology School of Medicine and Public Health University of Wisconsin Madison WI USA

**Keywords:** beam model, treatment planning

## Abstract

**Purpose:**

To explore candidate RayStation beam models to serve as a class‐specific template for a TrueBeam treatment delivery system.

**Methods:**

Established validation techniques were used to evaluate three photon beam models: a clinically optimized model from the authors’ institution, the built‐in RayStation template, and a hybrid consisting of the RayStation template except substituting average MLC parameter values from a recent IROC survey. Comparisons were made for output factors, dose profiles from open fields, as well as representative VMAT test plans.

**Results:**

For jaw‐defined output factors, each beam model was within 1.6% of expected published values. Similarly, the majority (57–66%) of jaw‐defined dose curves from each model had a gamma pass rate >95% (2% / 3 mm, 20% threshold) when compared to TrueBeam representative beam data. For dose curves from MPPG 5.a MLC‐defined fields, average gamma pass rates (1% / 1 mm, 20% threshold) were 92.9%, 85.1%, and 86.0% for the clinical, template, and hybrid models, respectively. For VMAT test plans measured with a diode array detector, median dose differences were 0.6%, 1.3%, and 1.1% for the clinical, template, and hybrid models, respectively. For in‐phantom ionization chamber measurements with the same VMAT test plans, the average percent difference was −0.3%, −1.4%, and −1.0% for the clinical, template, and hybrid models, respectively.

**Conclusion:**

Beam model templates taken from the vendor and aggregate results within the community were both reasonable starting points, but neither approach was as optimal as a clinically tuned model, the latter producing better agreement with all validation measurements. Given these results, the clinically optimized model represents a better candidate as a consensus template. This can benefit the community by reducing commissioning time and improving dose calculation accuracy for matched TrueBeam treatment delivery systems.

## 1 Introduction

Various resources are available to help guide the development of a new beam model in a treatment planning system. The TG‐106 report^1^ from the American Association of Physicists in Medicine (AAPM) provides information on the use of phantoms and detectors to acquire the measurement data typically used to generate a beam model. For TrueBeam treatment delivery systems (Varian Medical Systems, USA), multi‐institutional commissioning data have been reported and serve as a reference.^2^, ^3^, ^4^ Additionally, the AAPM has provided recommendations on end‐user beam model validation through reports for MPPG 5.a^5^ and TG‐119.^6^ Despite this commissioning guidance, articles reporting on dosimetry credentialing results from the Imaging and Radiation Oncology Core (IROC) have demonstrated difficulties in creating an accurate beam model, particularly for highly modulated plans.^7^, ^8^, ^9^ Furthermore, significant dose calculation differences have been noted when utilizing automated model generation based on closely corresponding beam commissioning data.^10^ In addition, with the prevalence of IMRT planning, multi‐leaf collimator (MLC) parameter values take on additional importance.^11^, ^12^

Historically, radiation oncology clinics have been required to create and validate unique machine‐specific beam models in their treatment planning system (TPS) due to variations in treatment delivery system (TDS) performance. In general, this process entails significant effort often under a compressed timeline, and this situation can be exacerbated in multi‐vendor environments. Starting with a template beam model may facilitate parameter value optimization, but significant effort is still required for validation and commissioning measurements. Nevertheless, with many newly arriving treatment machines meeting consistent performance specifications, the opportunity exists for use of an effective beam model template to potentially eliminate parameter value optimization and offer a reduced validation workload.

Historically, a TPS user often begins with published data as a starting place when constructing their own clinical beam model. With the advent of matched TrueBeam systems that satisfy Varian Enhanced Beam Conformance (EBC) specifications^13^, ^14^ for representative beam data, the use of template models may be considered even more tenable. Nevertheless, with a template‐based candidate beam model in place, it remains the responsibility of users to validate against a spectrum of test plans that match clinical practice to the extent that any present weaknesses are identified.

RayStation (RaySearch Laboratories, Sweden) MLC model parameter values have been shown to strongly affect its dynamic MLC delivery dose calculation. Multiple studies have analyzed these parameters for Elekta (Sweden) machines.^15^, ^16^, ^17^ Clinically used MLC parameter values have also been reported for the Varian Trilogy system.^18^, ^19^ For a TrueBeam treatment delivery system, Chen et al. presented a systematic approach for optimizing MLC parameter values based on IMRT QA dose measurements.^20^ Additionally, Saez et al. described a procedure using ionization chamber measurements of sweeping gap beams to determine RayStation parameters for both Millennium 120 and HD120 MLC systems.^21^ A recent publication by Glenn et al. reported a reference data set featuring the above RayStation MLC parameters as provided by clinical end‐users through an IROC survey for different treatment planning systems.^22^

The objective of this work is to use established validation techniques to identify the most optimal beam model from three candidates: a clinically optimized model from the authors’ institution (“clinical”), the built‐in RayStation template (“template”), and a hybrid based on the RayStation template except for substituting average parameter values from a recently published IROC survey (“hybrid”).^22^ We performed comparisons of measured versus calculated output factors, dose curves from jaw‐defined and MLC‐defined static treatment fields, as well as dose from representative VMAT test plans. By different TPS validation methods, we are able to discern performance differences between the three candidate beam models. This work demonstrates that it is not sufficient to simply use a vendor template or aggregate community data when building a model. The results in this work support the clinical model as a preferred candidate for a universally accepted template model with matched TrueBeam delivery systems.

## 2 MATERIALS AND METHODS

### 2.1 Candidate models

We created three different models in the RayStation 8B SP2 (v. 8.1.2.5) treatment planning system for a TrueBeam TDS equipped with the Millennium 120 MLC and satisfying EBC specifications.^13^ The first was clinically optimized model from the authors’ institution tuned according to locally developed methods. The second was the built‐in RayStation template model included in the TPS. The third was a hybrid model consisting of the RayStation template except substituting average parameter values from a recently published IROC survey.^22^ The beam models were assessed for flattened MV energies 6X, 10X, and 15X along with unflattened energies 6FFF and 10FFF. Relevant parameter values are shown in Tables 1, 2, 3. For the template and hybrid beam models, the normalization factor for each energy was adjusted such that the dose at 10 cm depth in water under TG‐51 conditions^23^ agreed with the existing clinical model. All TPS calculations were performed using a dose grid size of 1 mm within either the RayPhysics or RayPlanning module. Notably, RayStation 8B was the first software version to include a template model for 10X. Additionally, for the beam model parameters analyzed in this work, no changes were noted in template values between RayStation 6 through 8B.

**Table 1:** RayStation parameters for the clinical beam model from the authors’ institution

	6X	10X	15X	6FFF	10FFF
Primary X width (cm)	0.060	0.040	0.030	0.060	0.060
Primary Y width (cm)	0.045	0.070	0.030	0.060	0.075
MLC X Offset (cm)	0.026	0.029	0.027	0.020	0.022
MLC X Gain	0.0000	0.0000	0.0000	0.0000	0.0000
MLC X Curvature (cm^−1^)	0.00000	0.00000	0.00000	0.00000	0.00000
MLC Tip Width (cm)	0.360	0.310	0.180	0.400	0.450
MLC Transmission (%)	1.500	1.800	1.750	1.300	1.600
MLC TGW (cm)	0.040	0.040	0.040	0.050	0.050

**Table 2:** RayStation parameters for the template beam model from the built‐in TPS template

	6X	10X	15X	6FFF	10FFF
Primary X width (cm)	0.100	0.120	0.070	0.040	0.071
Primary Y width (cm)	0.112	0.080	0.069	0.040	0.071
MLC X Offset (cm)	0.000	0.060	0.000	0.000	0.000
MLC X Gain	0.0000	0.0030	0.0000	0.0000	0.0000
MLC X Curvature (cm^−1^)	0.00000	0.00000	0.00000	0.00000	0.00000
MLC Tip Width (cm)	0.200	0.187	0.200	0.200	0.200
MLC Transmission (%)	1.500	1.740	1.500	1.500	1.575
MLC TGW (cm)	0.040	0.050	0.040	0.040	0.040

**Table 3:** RayStation parameters for the hybrid beam model using the built‐in template beam model except substituting average parameter values from a recently published IROC survey.^22^

	6X	10X	15X	6FFF	10FFF
Primary X width (cm)	0.098	0.056	0.073	0.067	0.060
Primary Y width (cm)	0.080	0.077	0.055	0.083	0.070
MLC X Offset (cm)	0.016	0.010	0.000	0.015	0.030
MLC X Gain	0.0033	0.0100	0.0000	−0.0005	0.0000
MLC X Curvature (cm^−1^)	0.00052	0.00000	0.00040	0.00055	0.00000
MLC Tip Width (cm)	0.280	0.350	0.250	0.500	0.200
MLC Transmission (%)	1.604	1.280	2.015	1.047	1.370
MLC TGW (cm)	0.040	0.030	0.050	0.043	0.050

In the aforementioned IROC survey^22^ from 2020, user‐submitted values were provided for primary source size as well as a number of RayStation MLC parameters. The leaf tip width (LTW) is used to account for x‐ray transmission through the rounded end of an MLC. The tongue‐and‐groove width (TGW) accounts for transmission along exposed leaf sides defining an aperture edge. MLC positioning is accounted for as a function of field size using the terms offset, gain, and curvature which are polynomial coefficients in the expression:

(1) $$x_{\text{act}}=x_{\text{set}}+\text{OFFSET}+\text{GAIN}\cdot x_{\text{set}}+\text{CURVATURE}\cdot x_{\text{set}}^{2},$$

where $x_{\text{act}}$ represents the actual x‐ray field edge position in the dose distribution and $x_{\text{set}}$ is the MLC position value within the DICOM data file (for $x_{\text{set}}$ > 0). Finally, transmission through the full thickness of an MLC leaf is defined as $T_{\text{MLC}}$, while transmission through the MLC LTW and TGW zones is modeled as $\sqrt{T_{\text{MLC}}}$.

### 2.2 Jaw‐delineated beam analysis

Comparisons were made for output factors and dose profiles from jaw‐defined fields. Published values by Glide‐Hurst et al.^2^ at 100 cm source‐to‐surface distance (SSD) and 5 cm depth in water were used as reference values for field sizes from 3 × 3 cm^2^ to 30 × 30 cm^2^. Output factors in RayStation were calculated under the same setup conditions with a virtual water phantom.

In the comparison with measured percent depth dose (PDD) curves and lateral profiles, TrueBeam representative beam data^14^ provided by the vendor were used. These data were acquired at 100 cm SSD using a CC13 ionization chamber (IBA, Belgium).^3^ A custom Python script was used to perform local 1D gamma analysis with 2% / 3 mm criteria above a 20% dose threshold.^24^ Criteria were based on MPPG 5.a recommendations^5^ for basic dose profile comparisons given as ±2% locally in the high dose region with 3 mm distance‐to‐agreement in the penumbra region. Field sizes ranging from 3 × 3 cm^2^ to 30 × 30 cm^2^ were again evaluated with a calculation grid size of 1 mm. Dose profiles were assessed at water depths between 1.5 cm and 30 cm.

### 2.3 MPPG 5.a static beam analysis

In addition to beam model comparisons for jaw‐defined fields, an analysis was also performed for various MLC apertures following MPPG 5.a guidelines.^5^ Specifically, tests 5.4–5.8 and tests 7.1–7.2 from the report were executed utilizing MLC field definitions from Jacqmin et al.^25^ Field shapes included a small non‐rectangular aperture (5.4), MLC blocking over the central‐axis with a non‐aligned leaf bank (5.5), an off‐axis field featuring MLC blocking over the central‐axis using uniform leaf over‐travel (5.6), an asymmetric field at 80 cm SSD (5.7), a non‐rectangular field at oblique incidence (5.8), a 2 × 2 cm^2^ MLC‐defined field (7.1), and an irregular off‐axis MLC field plus a long on‐axis MLC field (7.2). The MPPG 5.a measurements were performed during TrueBeam commissioning at our institution using a Blue Phantom^2^ (IBA, Belgium) 3D water tank with a CC04 ionization chamber (IBA, Belgium) for tests 5.4–5.8 and a Razor diode (IBA, Belgium) for tests 7.1–7.2. With tests 5.4–5.8 and 7.1, inline profiles were acquired at depths of 3, 10, and 20 cm along with a crossline profile at 10 cm and a PDD curve. For each beam model in this study, a local gamma analysis was performed against these dose curve measurements with criteria of 2% / 3 mm and 1% / 1 mm above a 20% dose threshold using an open‐source MPPG 5.a Profile Comparison Tool^26^ in MATLAB (MathWorks, USA).

### 2.4 MPPG 5.a. VMAT diode array analysis

In the absence of vendor‐provided representative dose data for IMRT deliveries, the calculated dose from each RayStation beam model was compared to measurements using an EBC‐verified TrueBeam treatment delivery system with a Delta^4^ Phantom+ (ScandiDos, Sweden) for seven VMAT test plans. Four were geometry‐based per the AAPM TG‐119 test suite^6^ (small cylinder, large cylinder, C‐shape, and off‐axis cylinder), and three were anatomy‐based following institutional planning procedures for unilateral neck, chest wall, and lung sites. Diode detector spacing for the Delta^4^ phantom was 5 mm within a central 6 × 6 cm^2^ region of two orthogonal planar arrays and 10 mm outside of this region. A 3D global gamma analysis was performed with 3% / 2 mm criteria and a 10% dose threshold following AAPM TG‐218 recommendations regarding IMRT QA validation.^27^ Additionally, the median dose difference was assessed for each plan after corrections made for machine output, which were within 1% of the expected calibration value for each beam energy based on TG‐51 measurements in a water tank.^23^

### 2.5 MPPG 5.a. VMAT ionization chamber analysis

Using the institutional VMAT test suite described in Section 2.4, the calculated dose from each beam model was also compared to dose derived from ionization chamber measurements in a cylindrical Solid Water Tomo “cheese” phantom (Accuray, USA) with a TrueBeam treatment delivery system meeting EBC specifications. The absolute dose was calculated following the TG‐51 formalism.^23^ For each plan, the average dose calculated to an ionization chamber ROI in the TPS was compared to measurements using calibrated A1SL ionization chambers (Standard Imaging, USA) at six positions within the high dose region. Corrections were made for accelerator output deviations from the calibration setting (≤0.3% for flattened and ≤0.8% for FFF). For each beam energy and ionization chamber location, the local percent difference was determined between calculated and measured doses. The uncertainty in absolute dose from A1SL measurements was estimated as ±1%.^28^

## 3 RESULTS

### 3.1 Jaw‐delineated beam analysis

Table 4 contains output factors for each beam model determined at 100 cm SSD and 5 cm depth in water compared to measured values from Glide‐Hurst et al.^2^ Across all field sizes and models, calculated output factors were within 1.6% of expected published values. The average of individual differences from each corresponding measured output factor was 0.0% for each beam model. Comparing beam models in RayStation, the maximum spread between calculated values for a given field size was 1.3%.

**Table 4:** Calculated output factor (OF) values at 100 cm SSD and 5 cm depth in water for each beam model compared to published values from Glide‐Hurst et al.^2^

Energy	Field size (cm^2^)	Glide‐Hurst et al.^2^	Clinical		Template		Hybrid	
		OF	OF	% diff	OF	% diff	OF	% diff
6 MV	3 × 3	0.880	0.879	−0.1%	0.883	0.3%	0.883	0.3%
	6 × 6	0.949	0.946	−0.4%	0.948	−0.1%	0.948	−0.1%
	10 × 10	1.000	1.000	0.0%	1.000	0.0%	1.000	0.0%
	20 × 20	1.067	1.067	0.0%	1.067	0.0%	1.067	0.0%
	30 × 30	1.104	1.097	−0.7%	1.094	−0.9%	1.094	−0.9%
10 MV	3 × 3	0.885	0.892	0.8%	0.885	0.0%	0.886	0.1%
	6 × 6	0.952	0.957	0.5%	0.951	−0.1%	0.951	−0.1%
	10 × 10	1.000	1.000	0.0%	1.000	0.0%	1.000	0.0%
	20 × 20	1.056	1.060	0.4%	1.057	0.1%	1.057	0.1%
	30 × 30	1.086	1.087	0.1%	1.088	0.2%	1.088	0.2%
15 MV	3 × 3	0.878	0.887	1.0%	0.884	0.6%	0.884	0.6%
	6 × 6	0.958	0.956	−0.2%	0.955	−0.3%	0.955	−0.3%
	10 × 10	1.000	1.000	0.0%	1.000	0.0%	1.000	0.0%
	20 × 20	1.049	1.051	0.2%	1.047	−0.2%	1.047	−0.2%
	30 × 30	1.076	1.075	0.0%	1.074	−0.2%	1.074	−0.2%
6FFF	3 × 3	0.892	0.898	0.7%	0.896	0.4%	0.895	0.4%
	6 × 6	0.956	0.957	0.1%	0.957	0.1%	0.957	0.1%
	10 × 10	1.000	1.000	0.0%	1.000	0.0%	1.000	0.0%
	20 × 20	1.050	1.050	0.0%	1.046	−0.4%	1.046	−0.4%
	30 × 30	1.072	1.070	−0.2%	1.061	−1.0%	1.061	−1.0%
10FFF	3 × 3	0.921	0.924	0.3%	0.936	1.6%	0.936	1.6%
	6 × 6	0.973	0.970	−0.3%	0.977	0.4%	0.977	0.4%
	10 × 10	1.000	1.000	0.0%	1.000	0.0%	1.000	0.0%
	20 × 20	1.030	1.030	0.0%	1.026	−0.3%	1.026	−0.3%
	30 × 30	1.043	1.042	−0.1%	1.039	−0.4%	1.039	−0.4%
	*Avg*	−	−	0.0%	−	0.0%	−	0.0%
	*St Dev*	−	−	0.4%	−	0.5%	−	0.5%
	*Max*	−	−	1.0%	−	1.6%	−	1.6%
	*Min*	−	−	−0.6%	−	−1.0%	−	−1.0%

**Fig. 1:**
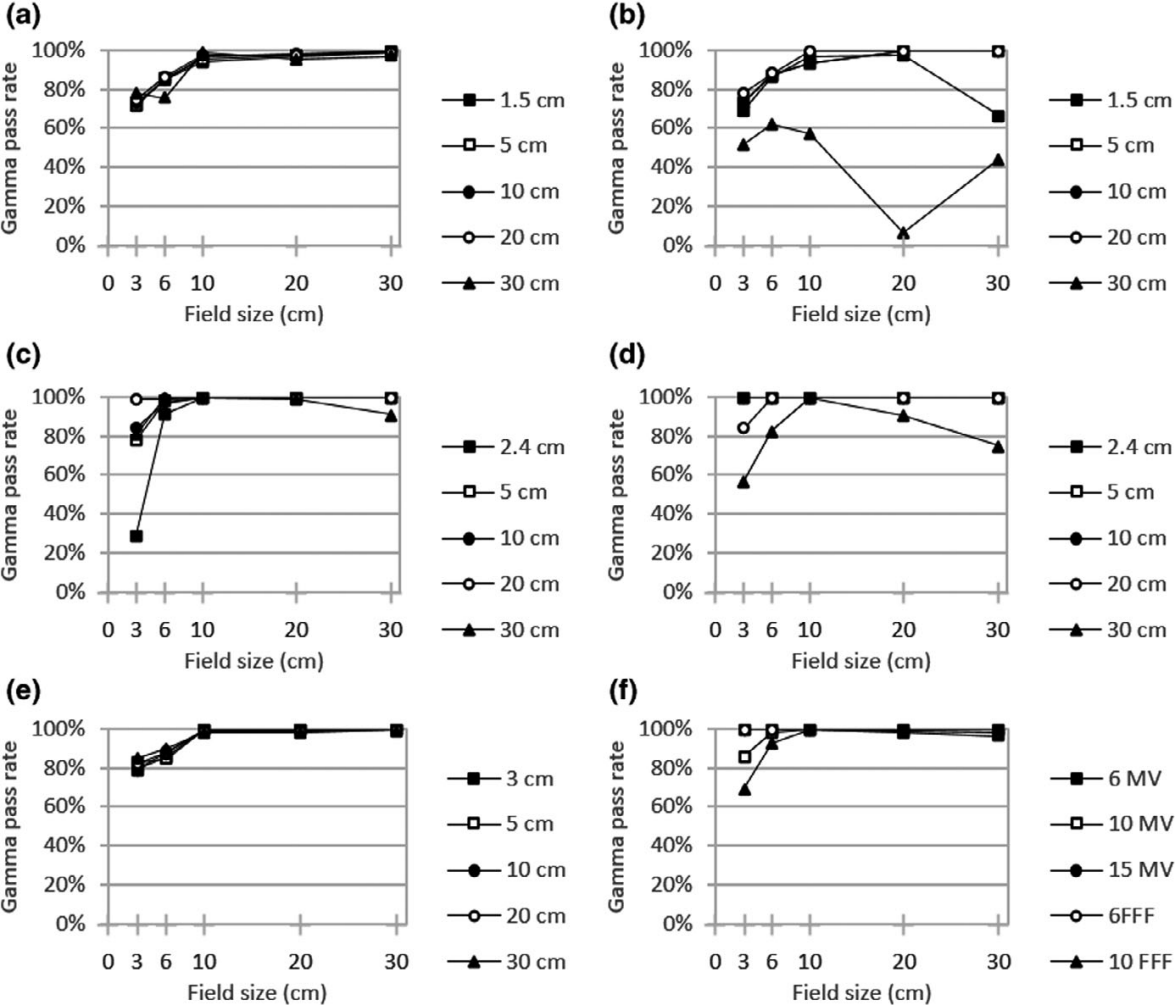
Local gamma analysis (2%/3mm and 20% threshold) for the clinical beam model versus TrueBeam representative beam data for jaw‐defined dose profiles at specified depths with (a) 6 MV (b) 6FFF (c) 10MV (d) 10FFF (e) 15MV and (f) PDD curves

**Fig. 2:**
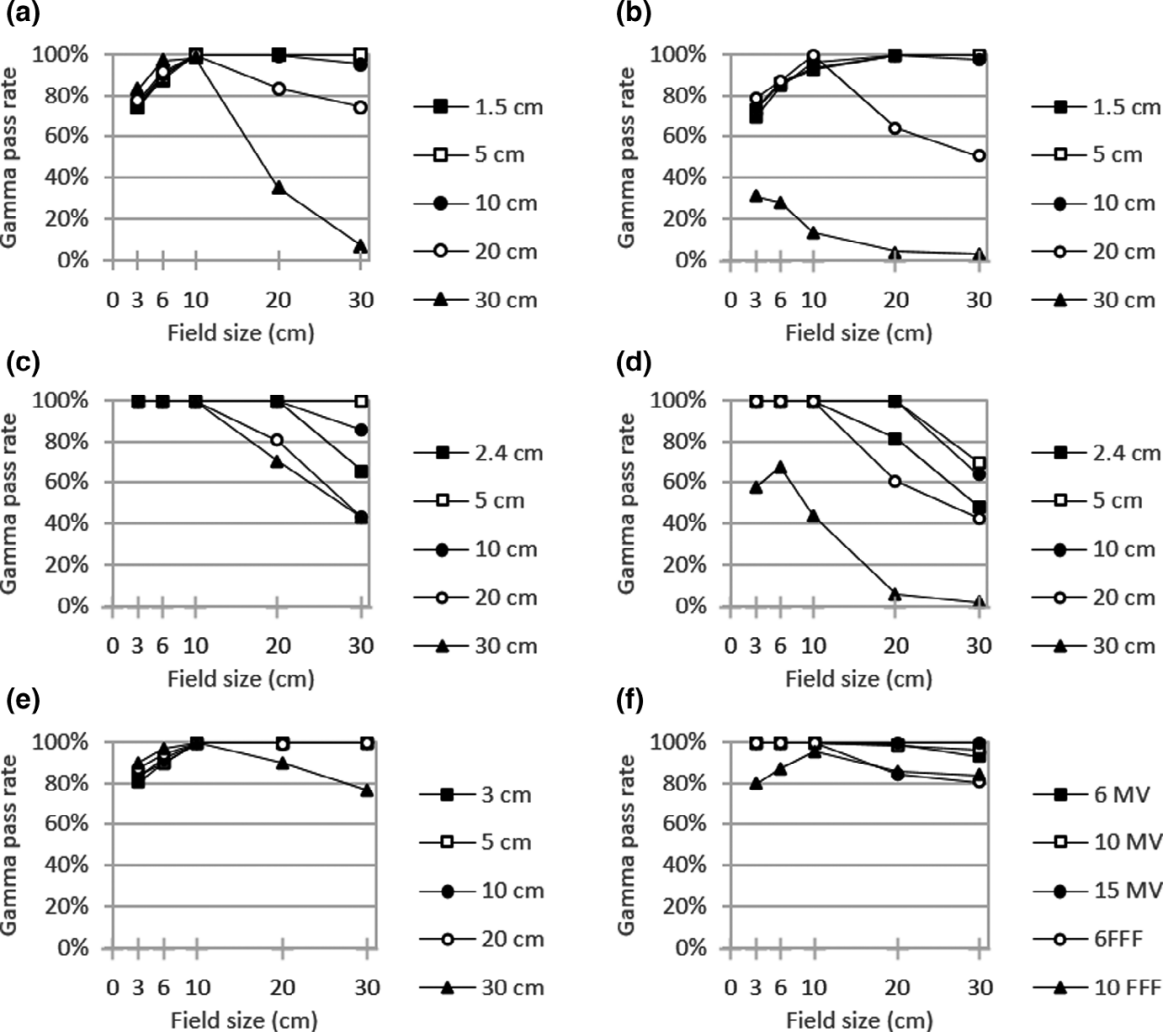
Local gamma analysis (2%/3mm and 20% threshold) for the template beam model versus TrueBeam representative beam data for jaw‐defined dose profiles at specified depths with (a) 6 MV (b) 6FFF (c) 10MV (d) 10FFF (e) 15MV and (f) PDD curves

**Fig. 3:**
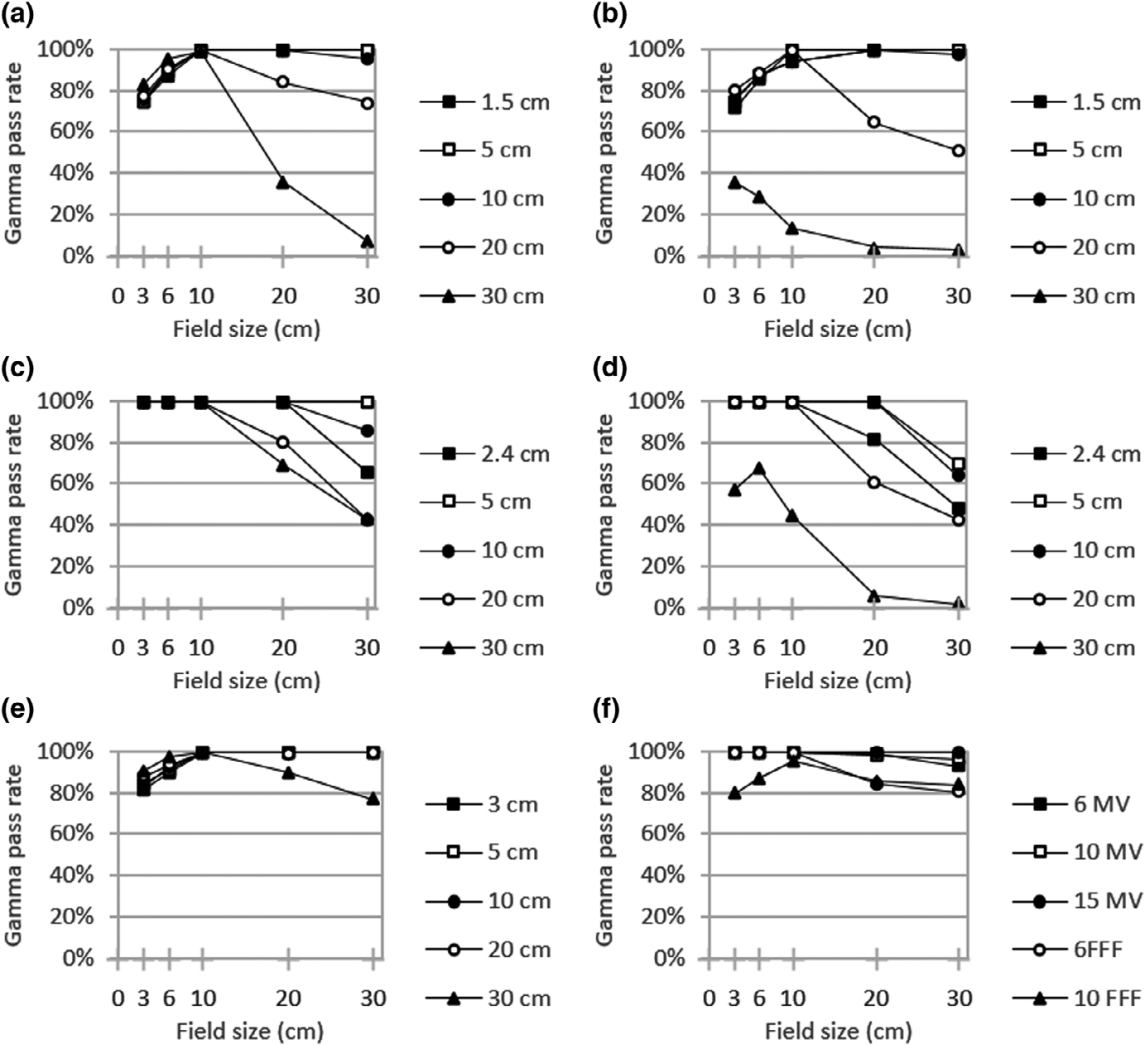
Local gamma analysis (2%/3mm and 20% threshold) for the hybrid beam model versus TrueBeam representative beam data for jaw‐defined dose profiles at specified depths with (a) 6MV (b) 6FFF (c) 10 MV (d) 10FFF (e) 15 MV and (f) PDD curves

Figures 1, 2, 3 display local gamma analysis results (2% / 3 mm and 20% threshold) for PDDs and lateral profiles from each beam model compared to TrueBeam representative beam data.^14^ For all combinations of beam energy and field size, the gamma pass rate was >95% for 99 of 150 (66.0%) profiles for the clinical beam model and 85 of 150 (56.7%) profiles for both the template and hybrid beam models. A gamma pass rate >90% was achieved for 107 of 150 (71.3%) profiles for the clinical model, 93 of 150 (62.0%) profiles for the template, and 95 of 150 (63.3%) profiles for the hybrid. Across all beam models, gamma pass rates averaged over all energies were highest for PDDs (95.3–97.4%) and dose profiles with a 10 × 10 cm^2^ field (93.5–96.9%). The lowest average gamma pass rate for each beam model was observed for profiles with either a 3 × 3 cm^2^ field (77.3% for clinical) or a 30 × 30 cm^2^ field (70.7% for both template and hybrid).

### 3.2 MPPG 5.a static beam analysis

**Fig. 4:**
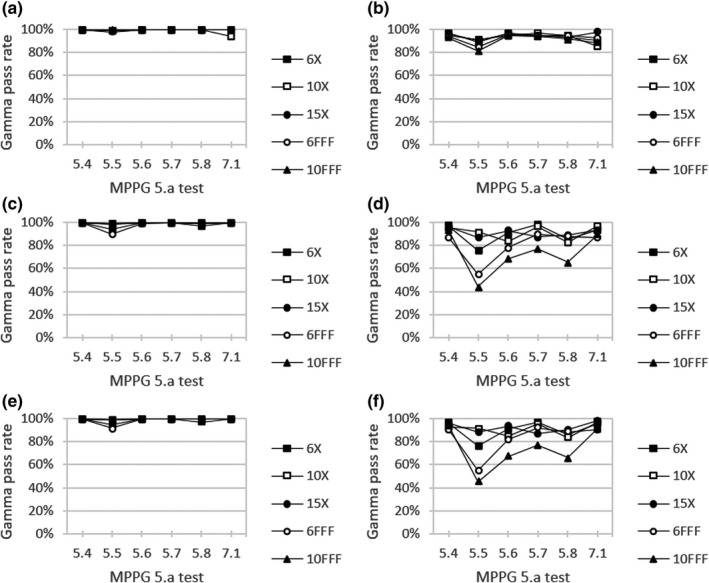
Local gamma analysis for (a,b) clinical, (c,d) template, and (e,f) hybrid beam models versus measured dose profiles for MPPG 5.a tests using citeria (left) 2%/3mm and (right) 1%/1mm with a 20% threshold

Figure 4 shows local gamma analysis results for each beam model versus measured dose profiles with MPPG 5.a test fields. Specifically, the gamma pass rate averaged over all dose profiles was assessed for each MPPG 5.a test and beam energy combination. All beam models performed well using local 2% / 3 mm criteria with pass rates >90%. In fact, the gamma pass rate averaged over all MPPG 5.a tests and energies was 99.5% for the clinical model, 99.0% for the template, and 99.2% for the hybrid. For the clinical beam model, the minimum pass rate (94.1%) was seen for MPPG 5.a test 7.1 with 10X. Minimum pass rates for both the template (90.1%) and hybrid (91.6%) beam models were observed for MPPG 5.a test 5.5 with 6FFF. Notable differences between beam models did become apparent when evaluating the same MPPG 5.a tests with stricter criteria of 1% / 1 mm. In this case, the average gamma pass rate for each beam model dropped to 92.9% for clinical, 85.1% for template, and 86.0% for hybrid. The minimum pass rate for each beam model was seen for MPPG 5.a test 5.5 and 10FFF with values of 81.4% for clinical, 44.2% for template, and 45.2% for hybrid.

Table 5 summarizes MPPG 5.a local gamma analysis results averaged across energy for a given beam model. Evaluating with 1% / 1 mm criteria, MPPG 5.a test 5.5 was found to give the lowest pass rate for clinical (93.1%), template (84.5%), and hybrid (83.3%). This test field consisted of a 15 × 20 cm^2^ outer aperture with MLC blocking over the central axis.^25^ MPPG 5.a test 5.6 had the next lowest pass rate for template (92.1%) and hybrid (89.2%). Test 5.6 was composed of a 5.5 × 15 cm^2^ MLC‐defined field centered 7.5 cm off‐axis.^25^ The remaining MPPG 5.a tests from this work were found to be relatively insensitive to detecting small changes between beam models with all gamma pass rates >93%.

**Table 5:** MPPG 5.a local gamma analysis results averaged across energy for each beam model using criteria 2% / 3 mm and 1% / 1 mm with a 20% threshold

MPPG 5.a test	Clinical		Template		Hybrid	
	2% / 3 mm	1% / 1 mm	2% / 3 mm	1% / 1 mm	2% / 3 mm	1% / 1 mm
5.4	99.8%	96.6%	100.0%	96.9%	100.0%	97.9%
5.5	99.8%	93.1%	99.5%	84.5%	99.7%	83.3%
5.6	99.8%	96.6%	99.6%	92.1%	99.6%	89.2%
5.7	100.0%	93.9%	100.0%	98.4%	100.0%	96.0%
5.8	99.9%	96.1%	99.3%	96.8%	99.9%	97.1%
7.1	100.0%	93.3%	100.0%	94.2%	100.0%	95.9%
*Avg*	99.9%	94.9%	99.7%	93.8%	99.9%	93.2%
*St Dev*	0.1%	1.7%	0.3%	5.1%	0.2%	5.8%
*Max*	100.0%	96.6%	100.0%	98.4%	100.0%	97.9%
*Min*	99.8%	93.1%	99.3%	84.5%	99.6%	83.3%

### 3.3 MPPG 5.a. VMAT diode array analysis

**Fig. 5:**
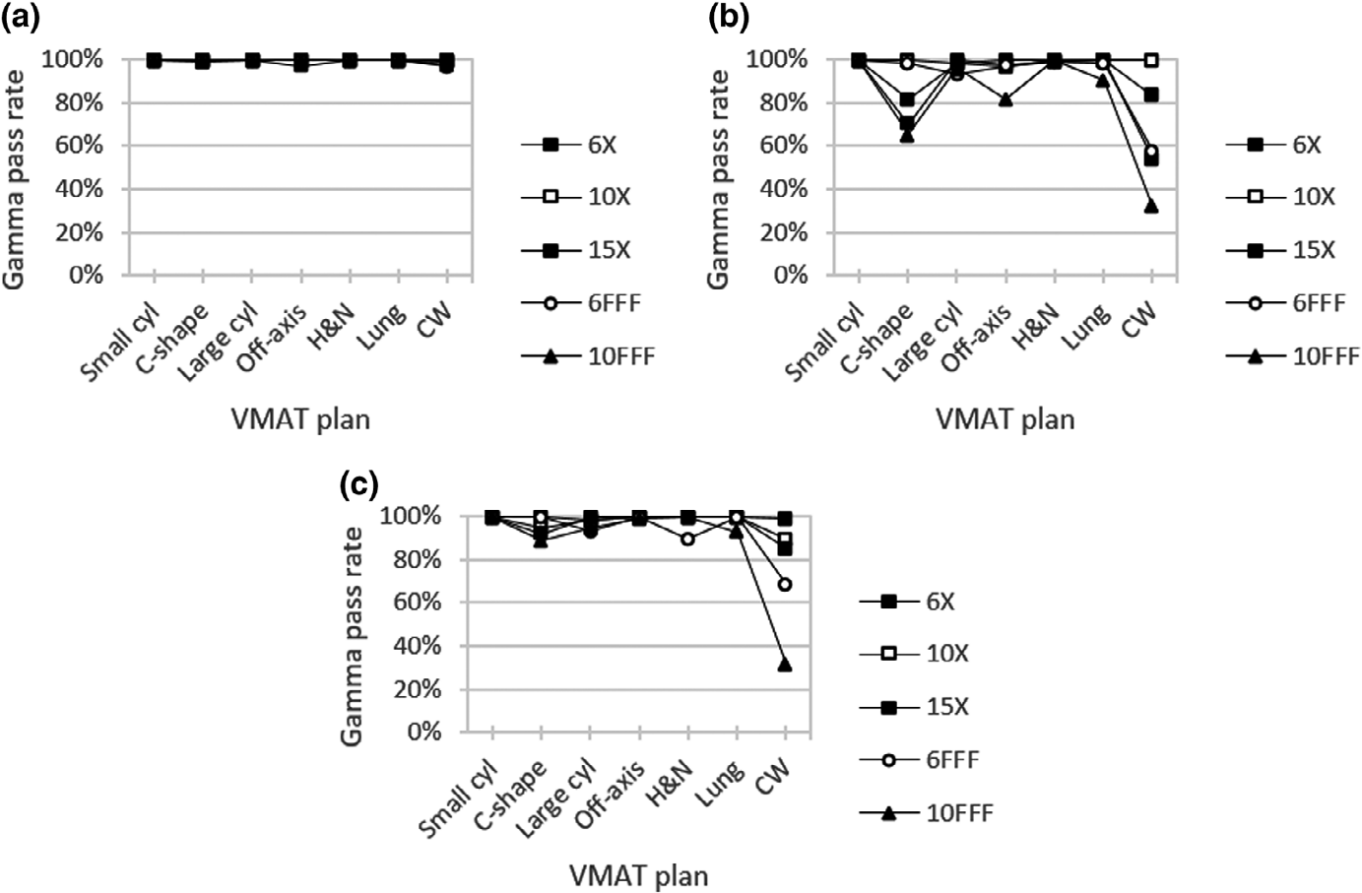
Global gamma analysis for (a) clinical, (b) template, and (c) hybrid beam models versus Delta^4^ Phantom+ measurements for VMAT test plans using criteria 3%/2mm with a 10% threshold

Figure 5 gives global gamma analysis results for each beam model versus Delta^4^ Phantom+ measurements for various VMAT test plans. For all beam energy and test plan combinations, the average gamma pass rate using 3% / 2 mm criteria and a 10% dose threshold was 99.6% for the clinical model, 91.0% for the template, and 94.6% for the hybrid. For each beam model, the lowest gamma pass rate was seen for the chest wall VMAT test plan. This minimum gamma pass rate was seen with 6FFF for clinical (96.5%) and with 10FFF for template (32.1%) and hybrid *(*31.5%).

Delta^4^ Phantom+ gamma results were compiled per plan in aggregate for all energies (Table 6). For each beam model, the lowest gamma pass rate was associated with the chest wall VMAT plan with values 65.3–98.6% using 3% / 2 mm criteria. The TG‐119 test plan for a C‐shape target was found to be the next‐most sensitive to beam model differences with a range of 16.7% between the minimum and maximum pass rates of 99.7% and 83.0%, respectively. Alternatively, the small cylinder plan was found to be the least challenging case for each beam model with gamma pass rates 99.8–100.0%. Gamma pass rates were greater than 94.6% for all other VMAT test plans with each beam model.

**Table 6:** Delta^4^ Phantom+ global gamma analysis results (3% / 2 mm, 10% threshold) and the median dose difference (MDD) averaged across energy for each beam model

VMAT plan	Clinical		Template		Hybrid	
	3% / 2 mm	MDD	3% / 2 mm	MDD	3% / 2 mm	MDD
Small cylinder	100.0%	−0.2%	99.8%	0.5%	100.0%	0.3%
Large cylinder	99.9%	0.0%	97.0%	0.4%	96.8%	0.4%
Off‐axis cylinder	99.4%	0.1%	94.6%	1.0%	99.7%	0.6%
C‐shape	99.7%	0.7%	83.0%	2.9%	95.0%	1.9%
Head & Neck	100.0%	0.8%	99.4%	0.8%	97.9%	0.7%
Lung	99.9%	0.4%	97.6%	0.1%	98.4%	0.3%
Chest wall	98.6%	2.0%	65.3%	3.8%	74.8%	3.2%
*Avg*	99.6%	0.6%	91.0%	1.3%	94.6%	1.1%
*St Dev*	0.5%	0.7%	12.7%	1.4%	8.9%	1.1%
*Max*	100.0%	2.0%	99.8%	3.8%	100.0%	3.2%
*Min*	98.6%	−0.2%	65.3%	0.1%	74.8%	0.3%

Table 6 also shows median dose differences for each VMAT test plan comparing Delta^4^ Phantom+ dose measurements to calculated dose values at each diode location. The clinical beam model gave the best agreement with a median dose difference of 0.6% averaged across all energies. Corresponding values were 1.3% for the template and 1.1% for the hybrid. Evaluating by plan type, the largest median dose difference for each beam model was seen with the chest wall VMAT plan with values 2.0–3.8%. All remaining median dose differences reported in Table 6 were ≤1.0%, except for the C‐shape target plan with the template and hybrid beam models at 2.9% and 1.9%, respectively.

### 3.4 MPPG 5.a. VMAT ionization chamber analysis

**Fig. 6:**
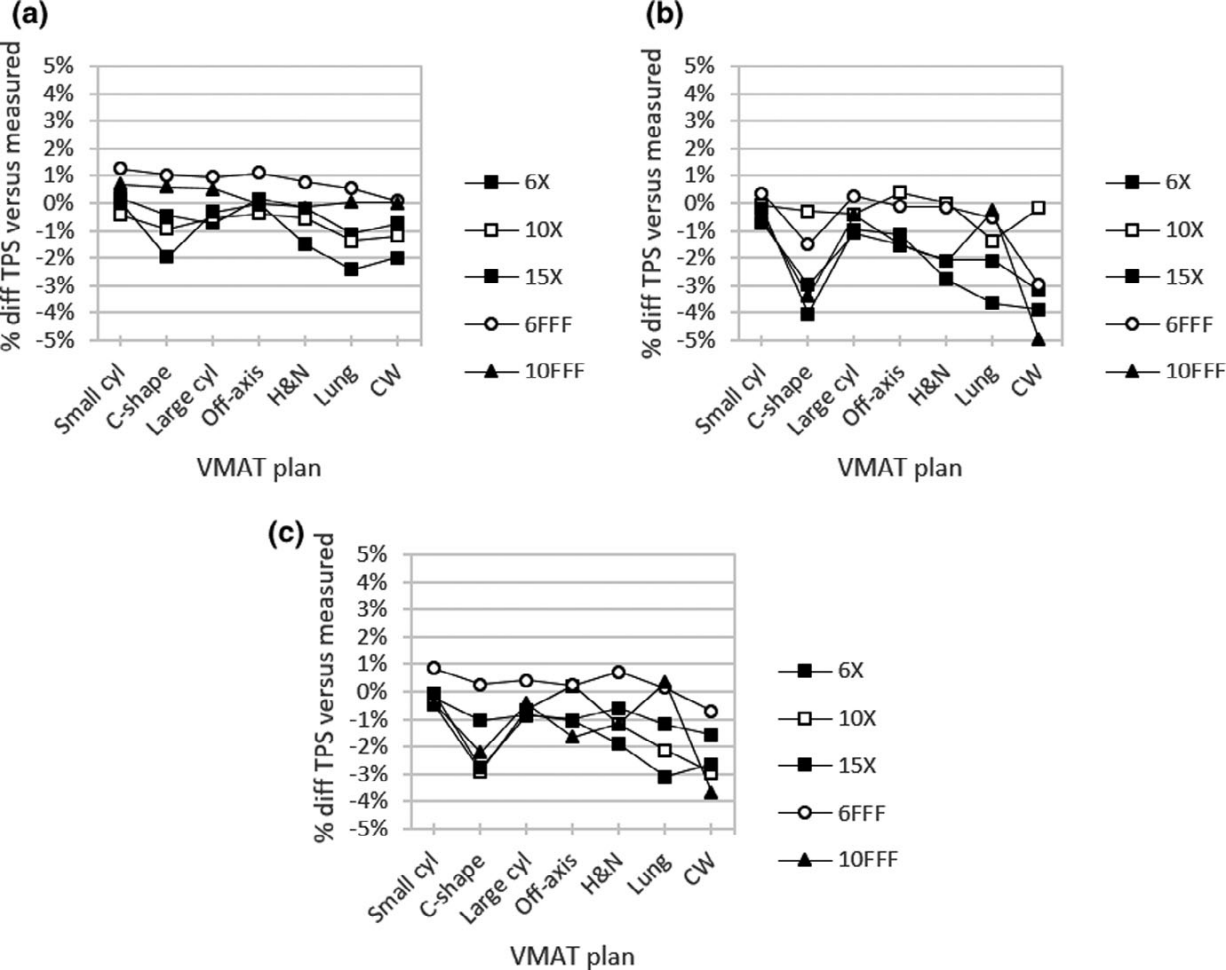
Percent difference for (a) clinical, (b) template, and (c) hybrid beam models versus in‐phantom ionization chamber measurements for VMAT test plans

Figure 6 presents percent dose differences between each beam model and output‐corrected ionization chamber measurements for the same VMAT test plans used in the Delta^4^ Phantom+ analysis. The clinical beam model showed the closest overall agreement with an average percent difference of −0.3% for all combinations of test plan and beam energy. Correspondingly, the average percent difference versus measurement data was −1.4% for template and −1.0% for hybrid. The largest deviation for the clinical beam model was −2.4% for the lung VMAT plan using 15X. For the remaining two beam models, the largest deviation was seen for the chest wall VMAT plan using 10FFF at −5.0% for template and −3.7% for hybrid. For instances in which the calculated dose was larger than the measured dose, the largest discrepancy for each beam model was 1.3% for the clinical model with the small cylinder TG‐119 plan and 6FFF, 0.4% for the template with the off‐axis TG‐119 plan and 10X, and 0.9% for the hybrid with the small cylinder plan and 6FFF.

Table 7 summarizes percent differences averaged across beam energy for calculated doses versus in‐phantom ionization chamber measurement of the VMAT test plans. Similar to the Delta^4^ Phantom+ results in Table 6, the chest wall plan gave the largest disagreement for the template (−3.0%) and hybrid (−2.3%) models along with the second‐largest disagreement for clinical (−0.8%). In this case, the largest average percent difference for the clinical beam model was −0.9% with the lung VMAT plan. The least challenging cases for each beam model were the small cylinder and large cylinder plans from TG‐119 with differences between −0.5% and 0.4%. The chest wall VMAT plan was found to be the most sensitive to beam model differences with a range of 2.3% between the minimum and maximum percent differences compared to in‐phantom ionization chamber measurements. The TG‐119 C‐shape target test plan had the next largest range at 2.1%. The large cylinder TG‐119 plan had the smallest range for the clinical (0.5%) and hybrid (0.1%) beam models, whereas the lung VMAT plan had the smallest range for the template (0.3%).

**Table 7:** Percent difference averaged across energy for each beam model versus in‐phantom ionization chamber measurements for VMAT test plans

VMAT plan	Clinical	Template	Hybrid
Small cylinder	0.4%	−0.2%	−0.1%
Large cylinder	−0.1%	−0.5%	−0.5%
Off‐axis cylinder	0.2%	−0.8%	−0.7%
C‐shape	−0.3%	−2.4%	−1.7%
Head & Neck	−0.3%	−1.4%	−0.8%
Lung	−0.9%	−1.6%	−1.2%
Chest wall	−0.8%	−3.0%	−2.3%
*Avg*	−0.3%	−1.4%	−1.0%
*St Dev*	0.5%	1.0%	0.8%
*Max*	0.4%	−0.2%	−0.1%
*Min*	−0.9%	−3.0%	−2.3%

## 4 Discussion

### 4.1 Model limitations and trade‐offs

The photon source parameter values for the clinical beam model were optimized with a primary focus on jaw‐defined fields under TG‐51 reference conditions.^23^ This included PDD curves and profiles for a 10 × 10 cm^2^ field and a depth of 10 cm. Based on RayStation model properties, this led to a trade‐off where the agreement was reduced with measurements for PDD sections and dose profiles at shallower and deeper depths. In particular, profiles were expected to disagree for large fields and deeper depths with a calculated penumbra that is artificially sharper due to the absence of kernel tilting within the RayStation dose engine. Furthermore, secondary source parameter values were set to produce output factor correction factors near unity as a function of field size, with the trade‐off being less agreement in profile fits at deeper depths and low shoulders away from the central axis. MLC parameters for the clinical model were initialized based on ray‐tracing analysis, and then the LTW was tuned based on ionization chamber measurements for representative VMAT test plans to produce the best agreement for targets 2–20 cm in diameter. In this way, the clinical model was fully optimized in the context of prioritized areas of agreement with regard to expected clinical use.

For the template beam model in RayStation, vendor documentation states that the included models may serve as a starting point but must also be validated during commissioning. Available materials do not make clear the extent to which these template models were optimized prior to release. Furthermore, areas of strength and weakness as a function of beam geometry are not well‐known based on included vendor documentation.

For the hybrid model, it was originally postulated that using average parameter values from a user poll would yield a community‐optimized model. Instead, the breadth of surveyed values indicated that clinical users have yet to reach a consensus for optimal RayStation parameters for matched TrueBeam machines. This is due at least in part to the fact that various TPS parameters are coupled. For example, MLC offset, tip width, and transmission all influence overall dose scaling for a dynamic MLC delivery, and different combinations of these parameter values can produce similar levels of overall dose agreement.

### 4.2 Test sensitivity

Jaw‐defined output factors (Table 4) demonstrated little sensitivity to the specific beam model differences evaluated in this work. This was expected with the only applicable parameters being the (X,Y) dimensions of the primary source, since changing the remaining MLC beam model values would have no impact on rectangular jaw fields. In this case, agreement within 1.6% was seen between calculated output factors and published measured values. The maximum coefficient of variation between published output factors by Glide‐Hurst et al.^2^ was similar at 1.2%. Furthermore, output factor values from the template and hybrid models were nearly identical despite changes in primary source width. This outcome suggests that output factor values are largely dependent on the photon energy spectrum and resulting output factor corrections, both of which were maintained when creating the hybrid model from the RayStation template model. Despite variation seen in output factor correction parameter values, all beam models produced reasonable agreement due to their direct connection with initial output factor measurements.

From the dose curve comparison for jaw‐defined fields (Figs. 1, 2, 3), certain trends emerged in the gamma analysis results across all models. In general, lateral profile agreement was better for intermediate field sizes, particularly a 10 × 10 cm^2^ field with average gamma pass rates of 93.5–96.9%. Conversely, profile agreement was typically worse for the largest field size of 30 × 30 cm^2^ (70.7% for both template and hybrid). This is somewhat expected since RayStation dose calculations do not incorporate kernel tilting, which would preferentially impact profile shapes for large fields and deeper depths. Additionally, for FFF beams, the agreement was generally worse since secondary source contributions were not available for the refinement of in‐field and out‐of‐field profile shape. For PDD curves, similar behavior was observed where the closest agreement with measured data occurred for a 10 × 10 cm^2^ field size (95.8–100.0% across beam models). The lowest gamma pass rates were associated with either a 3 × 3 cm^2^ field or a 30 × 30 cm^2^ field for each beam energy. This behavior comes about in part because the photon energy spectrum within RayStation is specified along the central axis and users can optimize bin weights to achieve the best agreement for an intermediate field (e.g. 10 × 10 cm^2^). Under this prioritization, low‐energy photon contributions from gantry scatter would be expected to contribute more to large field sizes while being attenuated by collimation with small fields. In addition, larger field profile agreement is affected by the kernel no‐tilt approximation in the RayStation dose algorithm implementation. The clinical model also prioritized flat output factor correction variation with jaw size over low‐shoulder profile agreement for larger field sizes, and this is borne out in the profile gamma behavior seen with field size.

The gamma analysis results for MPPG 5.a dose curves (Fig. 4 and Table 5) showed little sensitivity to small changes between beam models when using 2% / 3 mm criteria, since average pass rates were all >90%. For tighter criteria of 1% / 1 mm, larger differences emerged with the clinical beam model performing the best with an average pass rate of 92.9% compared to 85.1% for template and 86.0% for hybrid. In particular, the lowest pass rates for each beam model were seen for MPPG 5.a tests 5.5 and 5.6. Both of these test fields feature an off‐axis PDD, where dose calculation accuracy is limited by the lack of kernel tilting in RayStation as well as the fact that the photon energy spectrum is specified along the central axis for a 10 × 10 cm^2^ field. In this way, MPPG 5.a gamma analysis using 1% / 1 mm criteria was found to be a useful assessment highlighting performance differences between beam models with similar parameter values.

For the VMAT test plans assessed through diode array measurements (Fig. 5 and Table 6), beam model differences were highlighted only when using strict evaluation criteria. Compared to Delta^4^ Phantom+ measurements, the clinical beam model performed the best in this work with a median dose difference across all plans of 0.6% vs. 1.3% for the template and 1.1% for the hybrid. For in‐phantom ionization chamber measurements (Fig. 6 and Table 7) for the same VMAT test plans, the clinical model again showed the best agreement with an average percent difference of −0.3% vs. −1.4% for the template and −1.0% for the hybrid. All beam models performed well for the small cylinder and large cylinder TG‐119 plans, while differences in beam model accuracy up to 2.3% were apparent for more highly modulated deliveries, such as the chest wall and C‐shape VMAT plans. Previous studies^29^, ^30^, ^31^ have shown a correlation between lower IMRT QA pass rates and higher plan complexity using metrics such as MU‐weighted segment area, though the relation is not definitive with other groups reporting no such behavior.^32^, ^33^

For highly modulated treatment plans, small changes in an MLC parameter can propagate to produce detectable changes in absolute dose. Therefore, the performance of a beam model for representative treatment plans should be evaluated not just based on relative dose, but also the median difference when compared with absolute dose measurements. Absolute dose agreement for MLC‐modulated plans can provide information on MLC parameters that influence dose scaling in the plan.

In particular, for RayStation, an increased Leaf Tip Width parameter value leads to higher calculated plan dose. This is because a greater transmission value applies along a longer length of each MLC tip sweeping over a calculation point.^21^ Inversely, an increased Tongue Groove Width results in lower calculated plan dose. This is because each exposed MLC side is effectively wider, and transmission is reduced in the extended width. Correspondingly, it has been shown in Eclipse (Varian Medical Systems, USA) that using clinical plans to optimize MLC parameters can be more sensitive than static field profiles or dynamic leaf‐gap (DLG) measurements.^11^

Clinical users must keep in mind that the use of IMRT test plans for beam model validation requires the use of suitable calibrated absolute dosimetry equipment, and the output of the treatment machine should also be accounted for using a separate measurement setup, such as the TG‐51 protocol.^23^ As these results illustrate, the critical selection of clinical test plans for beam model validation must reflect the spectrum of treatments conducted by each center, and the representative test plans should be created using pre‐established planning protocols.

### 4.3 Best candidate beam model

In this work, the clinical beam model achieved the best or near‐best validation agreement across all analyses aggregated by test or beam energy. In coherence with established industry standards, these results further confirm that beam model optimization is necessary prior to effective clinical use. Importantly, since the clinical model was validated against a matched TrueBeam treatment delivery system, it is not unreasonable to consider that an institution employing the same beam model with a matched machine should be able to proceed directly to validation measurements without performing additional model parameter value optimization.

## 5 Conclusion

As it would broadly benefit the community, it is reasonable to pursue the construction of machine‐class templates that can be clinically validated by users without significant parameter adjustments. This work demonstrates this concept for a specific TPS‐TDS combination, namely RayStation and a Varian TrueBeam. Adoption of a reference beam model would require that the user’s treatment machine meets the same beam performance specifications for which the template model was built, and this assessment can be made during standard acceptance and commissioning measurements.

While this work evaluated a subset of existing candidate templates, it serves to highlight potential variations between vendor, communal average, and institutional beam models using established beam validation techniques. Our results indicate that beam model templates taken from the vendor and aggregate clinical results both represent reasonable starting points, but neither approach was as optimal as a clinically tuned model that produced better agreement with various validation measurements. We note that the vendor explicitly advises to not use the built‐in template clinically without additional validation, and as expected *a priori*, averages from an IROC user poll do not produce an optimal collection of co‐dependent parameter values to form a single usable model. Based on the results in this work, we propose a candidate RayStation reference model (“clinical”) for adoption by the community for this machine class. An accurate template model would be beneficial to clinical practice by reducing commissioning time and limiting errors during the development of a complex model.

## Conflict of Interest

The authors have no relevant conflict of interest to disclose.

## Data Availability

The data that support the findings of this study are available from the corresponding author upon reasonable request.
